# Correction: Evaluation of the predictive ability of ultrasound-based assessment of breast cancer using BI-RADS natural language reporting against commercial transcriptome-based tests

**DOI:** 10.1371/journal.pone.0229584

**Published:** 2020-02-19

**Authors:** Neema Jamshidi, Jason Chang, Kyle Mock, Brian Nguyen, Christine Dauphine, Michael D Kuo

The first author’s name is spelled incorrectly. The correct name is: Neema Jamshidi. The correct citation is: Jamshidi N, Chang J, Mock K, Nguyen B, Dauphine C, Kuo MD (2020) Evaluation of the predictive ability of ultrasound-based assessment of breast cancer using BI-RADS natural language reporting against commercial transcriptome-based tests. PLoS ONE 15(1): e0226634. https://doi.org/10.1371/journal.pone.0226634
