# Supplementary material for: Evaluation of the predictive ability of ultrasound-based assessment of breast cancer using BI-RADS natural language reporting against commercial transcriptome-based tests
Source: PLoS One. 2020 Jan 10;15(1):e0226634. doi: 10.1371/journal.pone.0226634 (PMC6953781; doi:10.1371/journal.pone.0226634)
Supplement: Attachment — Submitted filename: comments.1.pdf [file pone.0226634.s006.pdf]

We thank the reviewers for their evaluation of the manuscript and constructive comments. The manuscript has been revised to reflect the changes that are described below.

Reviewer #1: All comments have been addressed. No further comments. I think this will be a valuable manuscript.
Thank you for your constructive comments and revisions!

Reviewer #3: This is an interesting look at the breast US features, as they correlate with breast cancer prognostic and predictive markers. It is nicely presented.
Thank you for your comments and re-review.

However, I do take issue with the determination, under the method section, that " AUC of 0.6 to be considered as a supportive adjunct to help guide the decision of whether a molecular based tissue test is warranted"(page 37). I am not sure how this conclusion was reached, since this is not a study assessing the need for employing a prognostic marker, but rather a study looking at correlating a prognostic marker with imaging features. The decision on when to use a prognostic marker resides with the oncologist and is influenced by many patient and tumor characteristics and I do not believe it can be made on imaging features alone, at this point in time.
The purpose of the statement in the Methods was simply to describe our pre-defined criteria at the outset of the study. The main point was to state that an AUC < 0.9 would be too weak to expect it to develop into any surrogate for molecular phenotypes, but that an AUC that was > 0.6, although not strong enough to become a surrogate, could potentially identify features of interest. It appears that the language that we used was objectionable, so we have revised this and hopefully it is more palatable for the Reviewer in the current revision.

The conclusion that the US features employed here can be used to predict when to use a prognostic marker is, therefore, far reaching and should be revised.
We were careful to avoid making definitive statements and in the text stated that the ultrasound features have the potential to be used as screening tests (not for the purpose of exclusion, but rather to support the need for such tests). Still to address the reviewer's concern, we have deleted the final sentence of the conclusion that refers to the potential to adjust pre-test probabilities.

What this study demonstrates is a correlation between the US features and Oncotype DX and, to a lesser degree, to the MammaPrint, but the correlation is moderate at best. Therefore, it can not be used in place of these tests to predict who needs chemotherapy.
We agree that the ultrasound features should not be used in place of transcriptomic tests. At no point in the original or revised manuscripts did we state or imply this – in fact we argue the opposite and in the Discussion we (gently) draw attention to studies that have argued for replacing transcriptomic tests with correlations that are < 0.8. We believe that lending such weight to the imaging studies is overzealous at best and this is why we see value in the ultrasound test as a 'supportive' test and not a direct surrogate (the features can be assessed with essentially no additional cost, since ultrasound evaluation is generally part of the workup for a breast mass, and it can help justify the acquisition of the transcriptomic test). We note that further studies are needed to validate this prospectively and in a larger cohort.
In an age where physician recommendations and requests are at the mercy of insurance companies, an unfortunate reality of the situation is that depending on the insurance carrier and the cost of a test, patients may or may not be able to have a test, even if the primary oncologist and everyone at the institutions Tumor Board believes that the test is warranted. Thus an important potential application for non-invasive imaging tests is the potential to provide additional information that may point to the utility of acquiring one of the transcriptomic tests; we think it is worth mentioning the possible application of ultrasound descriptors, without overstating their utility at this time.

The study is promising in that it shows a correlation and I agree with the conclusion that adding other imaging features to the ones evaluated in this study may improve the AUC and hopefully achieve the >0.9 AUC.
Thank you, we hope to be exploring these features in future studies.

The Discussion is very lengthy, as mentioned by another reviewer. I would consider moving some of the discussion under the introduction part, for example the costs of MRI and prognostic markers and focus the discussion on the findings of this study and how it compares to other studies, like you did under the Conclusion section.
We curtailed some of the Discussion in the last revision, currently it is less than twice the length of the Introduction, which we believe is well within the norm for printed as well as electronic journals.
