# Supplementary material for: Evaluation of the predictive ability of ultrasound-based assessment of breast cancer using BI-RADS natural language reporting against commercial transcriptome-based tests
Source: PLoS One. 2020 Jan 10;15(1):e0226634. doi: 10.1371/journal.pone.0226634 (PMC6953781; doi:10.1371/journal.pone.0226634)
Supplement: Attachment — Submitted filename: responses.1.docx [file pone.0226634.s005.docx]

We thank the reviewers for their evaluation of the manuscript and constructive comments. The manuscript has been revised to reflect the changes that are described below.

Reviewer #1: Summary:
This is an interesting paper that explores an aspect of radiogenomics. I think it certainly adds to the current literature on this subject.
Thank you, we too believe that this initial evaluation may prompt further studies to extract further information from ultrasound studies, with the potential to serve as useful adjuncts in motivating the need for tissue based transcriptome studies, in the appropriate cohorts.

Introduction:
1) Consider changing the first paragraph to focus more on the value of US in breast cancer. It is too generic for the focus of this paper.
This is a great point, since the focus of the manuscript is on ultrasound. We have added some additional references and statements highlighting the role and relevance of ultrasound.

Methods:
2) It would be important to know what reporting system is used for US …is it powerscribe where the radiologist can describe any terminology they want (compliant with BIRADS or not) or is it a program like MagView where the radiologist is somewhat forced to use BIRADS terms?

There were template reports, however a dictation system was used (Powerscribe, Nuance). All of the reports used BI-RADS terminology (as provided in the Supporting Information).

3) Need to mention how many radiologists reported on US and what was their experience in terms of years.
The studies were read by three sub-specialty trained radiologists (all with at least 4 years of experience). Methods were updated to reflect this.

4) 4th paragraph of methods: capitalize where appropriate
Done.

Discussion
5) I am not sure the entire discussion on costs is applicable. It is ok to mention it in a few sentences but such a long section seems out of scope for this paper.
We have abbreviated the paragraph regarding the cost benefits and trade-offs.

Reviewer #2: The premise of this study is very intriguing and I would recommend publication after consideration of minor revisions. It is clear that while this is an interesting study with potentially very interesting implications, substantially more data from a very large, multicenter, prospective trial, will be needed to be confident that an AUC of 0.6 truly provides sufficient accuracy for ultrasound features to be considered a supportive adjunct to guide the decision of whether molecular based tissue testing is warranted. The transcriptome tests guide critical clinical decisions regarding the benefit of administering vs. not administering chemotherapy and stratify high risk patients from lower risk patients. A significantly greater level of confidence from a much larger study(ies) will be needed before imaging characteristics of tumors can influence clinical decisions about whether to use a tissue-based tests to clarify the need for chemotherapy. While the authors do not purport to draw a definite conclusion, more definitive language from the authors about the limitations of this study, which in essence provides preliminary data, and the need for additional robust data is strongly recommended.

Thank you for your comments and review of the manuscript.

We have added additional text to highlight the fact that additional studies with larger cohorts will be needed to further test the potential utility of these features (particularly multi-institutional, given the potential for imager-dependent variability in ultrasound exams).

We feel it is worth mentioning however, given that the transcriptomic tests are dependent on insurance company approval and the ability of a patient to have the test is driven in large part by whether they have “good” or “bad” insurance, we feel that any additional data (especially if there is no additional cost associate with it) that can help indicate potential benefit from the transcriptomic test provides some value (especially considering some published studies promoting MR based “radiogenomic” predictors compared to transcriptomic tests have argued for the role of surrogacy with AUCs < 0.8 – to that end, we feel that an AUC ~ 0.7 for an adjunctive test is reasonable).
